# Variation in C - reactive protein response according to host and mycobacterial characteristics in active tuberculosis

**DOI:** 10.1186/s12879-016-1612-1

**Published:** 2016-06-10

**Authors:** James Brown, Kristina Clark, Colette Smith, Jennifer Hopwood, Oliver Lynard, Michael Toolan, Dean Creer, Jack Barker, Ronan Breen, Tim Brown, Ian Cropley, Marc Lipman

**Affiliations:** Department of Respiratory Medicine, Royal Free London NHS Foundation Trust, London, UK; Department of Infection and Population Health, University College London, London, UK; UCL Respiratory, Division of Medicine, University College London, London, UK; Department of Respiratory Medicine, Guys and St Thomas’ NHS Foundation Trust, London, UK; Department of Respiratory Medicine, King’s College Hospital NHS Foundation Trust, London, UK; National Mycobacterial Reference Laboratory, London, UK

**Keywords:** Tuberculosis, C-reactive protein, Acute phase response, Innate immune response

## Abstract

**Background:**

The C - reactive protein (CRP) response is often measured in patients with active tuberculosis (TB) yet little is known about its relationship to clinical features in TB, or whether responses differ between ethnic groups or with different *Mycobacterium tuberculosis* (M.tb) strain types. We report the relationship between baseline serum CRP prior to treatment and disease characteristics in a metropolitan population with TB resident in a low TB incidence region.

**Methods:**

People treated for TB at four London, UK sites between 2003 and 2014 were assessed and data collected on the following characteristics: baseline CRP level; demographics (ethnicity, gender and age); HIV status; site of TB disease; sputum smear (in pulmonary cases) and culture results. The effect of TB strain-type was also assessed in culture-positive pulmonary cases using VNTR typing data.

**Results:**

Three thousands two hundred twenty-two patients were included in the analysis of which 72 % had a baseline CRP at or within 4 weeks prior to starting TB treatment. CRP results were significantly higher in culture positive cases compared to culture negative cases: median 49 mg/L (16–103 mg/L) vs 19 mg/L (IQR 5–72 mg/L), *p* = <0.001. In those with pulmonary disease, smear positive cases had a higher CRP than smear negative cases: 67 mg/L (31–122 mg/L) vs 24 mg/L (7–72 mg/L), *p* < 0.001. HIV positive cases had higher baseline CRPs than HIV negative cases: 75 mg/L (26–136 mg/L) vs 37 mg/L (10–88 mg/L), *p* <0.001. Differing sites of disease were associated with differences in baseline CRP: locations that might be expected to have a high mycobacterial load (e.g. pulmonary disease and disseminated disease) had a significantly higher CRP than those such as skin, lymph node or CNS disease, where the mycobacterial load is typically low in HIV negative subjects. In a multivariable log-scale linear regression model adjusting for host characteristics and M.tb strain type, infection with the East African Indian strain was associated with significantly lower baseline-CRP (fold-change in CRP 0.51 (0.34–0.77), *p* < 0.01).

**Conclusions:**

Host and mycobacterial factors are strongly associated with baseline CRP response in tuberculosis. This analysis suggests that there are important differences in innate immune response according to ethnicity, Mtb strain type and site of disease. This may reflect differing mycobacterial loads or host immune responses.

**Electronic supplementary material:**

The online version of this article (doi:10.1186/s12879-016-1612-1) contains supplementary material, which is available to authorized users.

## Background

The serum C-reactive protein (CRP) concentration is commonly measured during the investigation or treatment of active Tuberculosis (TB) [1]. Relatively little is known about how different disease states (e.g. site of disease), host factors (age, gender, and ethnicity) or mycobacterial characteristics (strain type) might influence CRP values. Furthermore, as the CRP can be taken as an indicator of the acute innate immune response, such data may offer clues to the differences in this response according to host and mycobacterial characteristics.

CRP acts as part of the innate immune response by binding to ligands such as phosphocholine on dead or dying cells and bacteria, activating complement C1q and the classical complement pathway and promoting phagocytosis. It is primarily produced in the liver (although some tissue production occurs) in response to Il-6, (plus Il-1β and TNFα) secretion, the major source of which is macrophages. It is genetically ancient and highly conserved [2]. Its relatively short half-life (around 19 hours) means that serum CRP levels reflect the rate of production and can therefore act as a biomarker of disease activity.

Several studies have evaluated the utility of CRP testing in TB, demonstrating a lower median CRP in TB compared to bacterial pneumonia [3] and that the addition of CRP testing to clinical assessment can aid the management of HIV positive patients in a high TB incidence setting [4–6]. Furthermore high baseline CRP levels have been shown to correlate with slow sputum culture conversion [7] and with poor treatment outcomes;[8] and a raised CRP after 8 weeks of TB treatment has been found to be predictive of continued culture-positive status[9]. Different TB strain-types are believed to have co-evolved with different human populations [10], and mycobacteria from different M.tb lineages can provoke different inflammatory responses in human macrophages in-vitro [11]. In addition, differences in inflammatory responses in active Tuberculosis have been reported in different ethnic groups [12].

The extent to which these possible effects impact on measurements of inflammatory response seen in clinical practice is not known, as the distribution of CRP results in active TB, and how clinical parameters may affect this has not been explored in a large patient group. We therefore investigated a cohort of individuals being treated for active TB and examined the association between the baseline CRP results and host and mycobacterial characteristics.

## Methods

We undertook a retrospective study of a cohort of patients treated for active TB between 2003 and 2014 in four hospitals in London, UK - a setting with an incidence of TB of around 35–40 cases/100,000 pa during the study period [13]. Active TB was defined as any patient treated for TB. CRP results at the time of, or within 4 weeks prior to, TB treatment initiation were obtained from hospital electronic patient records. These were measured by the clinical biochemistry laboratories in the treating hospitals, using standard immuno-turbidimetric assays. The normal range for the CRP at all sites was 0–5 mg/L; there were no differences in this according to subject age, ethnicity or gender. Some laboratories did not report exact values for CRP results within the normal range (instead giving the result as being below 5 mg/L) in these cases a value of 4 mg/L was used in the analysis.

Data were collected from existing hospital databases and electronic patient records, supplemented where necessary by information from the London TB Register, an electronic database which forms part of Public Health England’s Enhanced TB surveillance system. We obtained information on the following parameters: baseline CRP (when, or within four weeks before, starting treatment); patient characteristics (age, gender and ethnicity); site of disease; smear and culture results. We also collected data on M.tb strain-type for a subgroup of isolates from patients with pulmonary TB.

Data were analysed in Excel (Microsoft) and SPSS (IBM). Descriptive statistics of characteristics associated with baseline CRP were calculated. Multivariable linear regression models were built, using the log-CRP (which approximated a normal distribution). As well as a subgroup analysis evaluating the effect of M.tb strain type, sensitivity analyses were undertaken using only culture-confirmed TB cases and with alternative coding using place of birth as a proxy for ethnicity.

## Results

Data regarding 3,222 patients treated for active TB were obtained. Of these, 2307 (72 %) had a CRP result which we could include in the analysis. Patients were more likely to have a CRP measured if they had culture positive disease compared to culture negative (74 % with a CRP result vs 65 %, *p* <0.001), HIV positive compared to HIV negative (81 vs 77 %, *p* < 0.001) and if they were male (67 vs 63 %, *p* = 0.035) (Additional file 1: Table S1). The CRP was less likely to be measured in those of White ethnicity compared to other ethnic groups (59 vs 72 %, *p* <0.001) or who had disease at peripheral lymph node or CNS sites (62 and 66 % respectively, compared to 71–90 % at other disease sites (*p* <0.001).

Characteristics of the patients included in the analysis are presented in Table 1. The median age was 37 years (IQR 25–47 years), 55 % had pulmonary disease, 67 % were culture positive and 10 % HIV co-infected. For the whole cohort, the median CRP was 40 mg/L (IQR 11–94 mg/L) with a strongly positively skewed distribution. Thirteen percent of the whole cohort and 9 % of those with culture-confirmed disease had a baseline CR*p* <5 mg/L.

**Table 1:** Cohort characteristics

Total cohort (*N* = 3,222)		N (%)
Age, years	<16	243 (7.6 %)
	16–34	1482 (46.4 %)
	35–50	831 (26 %)
	50+	635 (19.9)
Gender male	Male	1466 (57.5 %)
	Female	1078 (42.3 %)
Ethnicity code	White	616 (23.0 %)
	Black African/Caribbean	977 (36.5 %)
	Asian	641 (23.9 %)
	Eastern Mediterranean and North Africa	297 (11.1 %)
	Americas	148 (5.5 %)
Site of disease	Pulmonary or mediastinal lymph node	1780 (55.2 %)
	Pleural or pericardial	233 (7.2 %)
	Abdominal	119 (3.7 %)
	Miliary/disseminated	75 (2.3 %)
	Bone and joint	197 (6.1 %)
	Peripheral lymph node	533 (16.5 %)
	Skin and soft tissue	31 (1.0 %)
	CNS	126 (3.9 %)
	Other/unknown	128 (4.0 %)
Culture status	Culture negative	1027 (32.5 %)
	Culture positive	2136 (67.5 %)
Smear status (pulmonary cases)	Smear negative	598 (54.7 %)
	Smear positive	495 (45.3 %)
HIV Status	HIV positive	333 (10.3 %)
	HIV negative	2020 (62.8 %)
	HIV status unknown	865 (26.9 %)

**Fig. 1:**
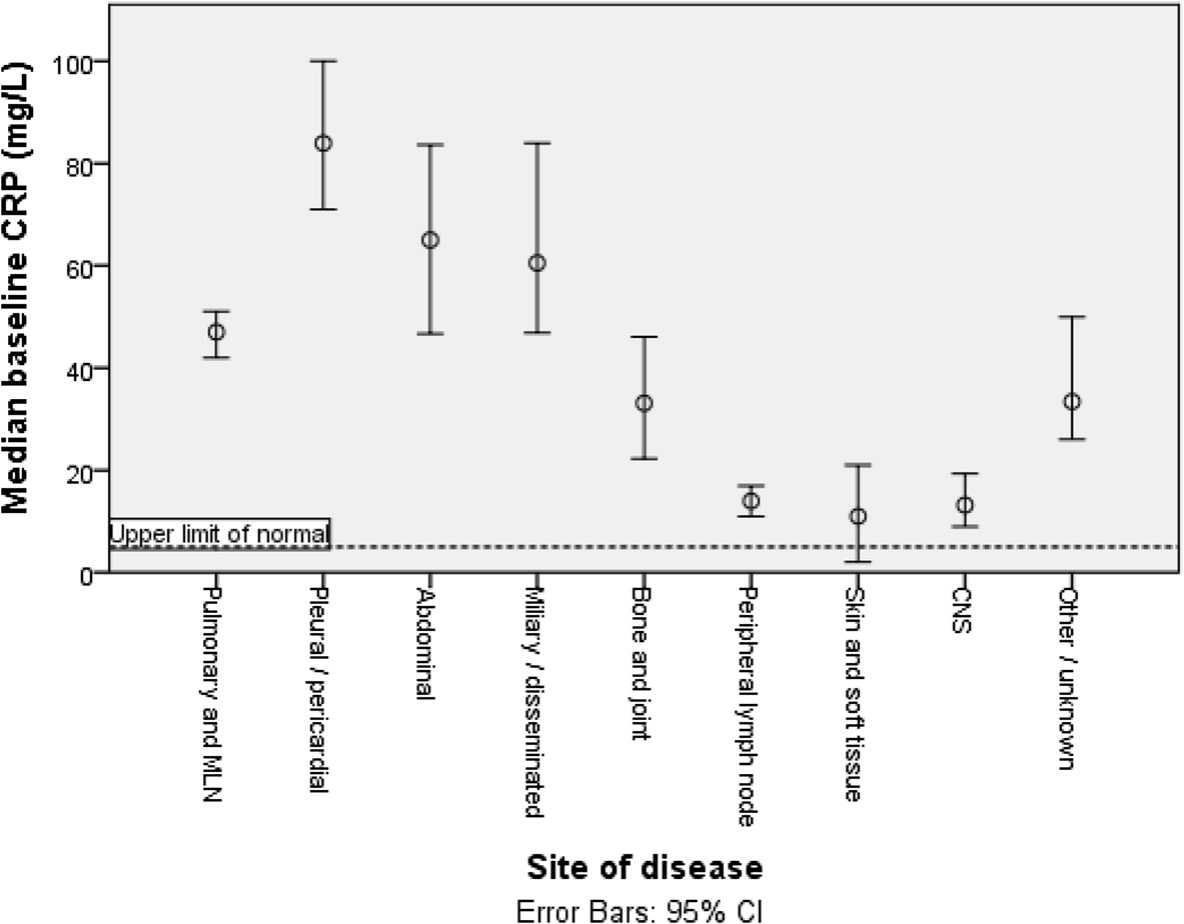
Association between sites of disease and median baseline CRP

**Fig. 2:**
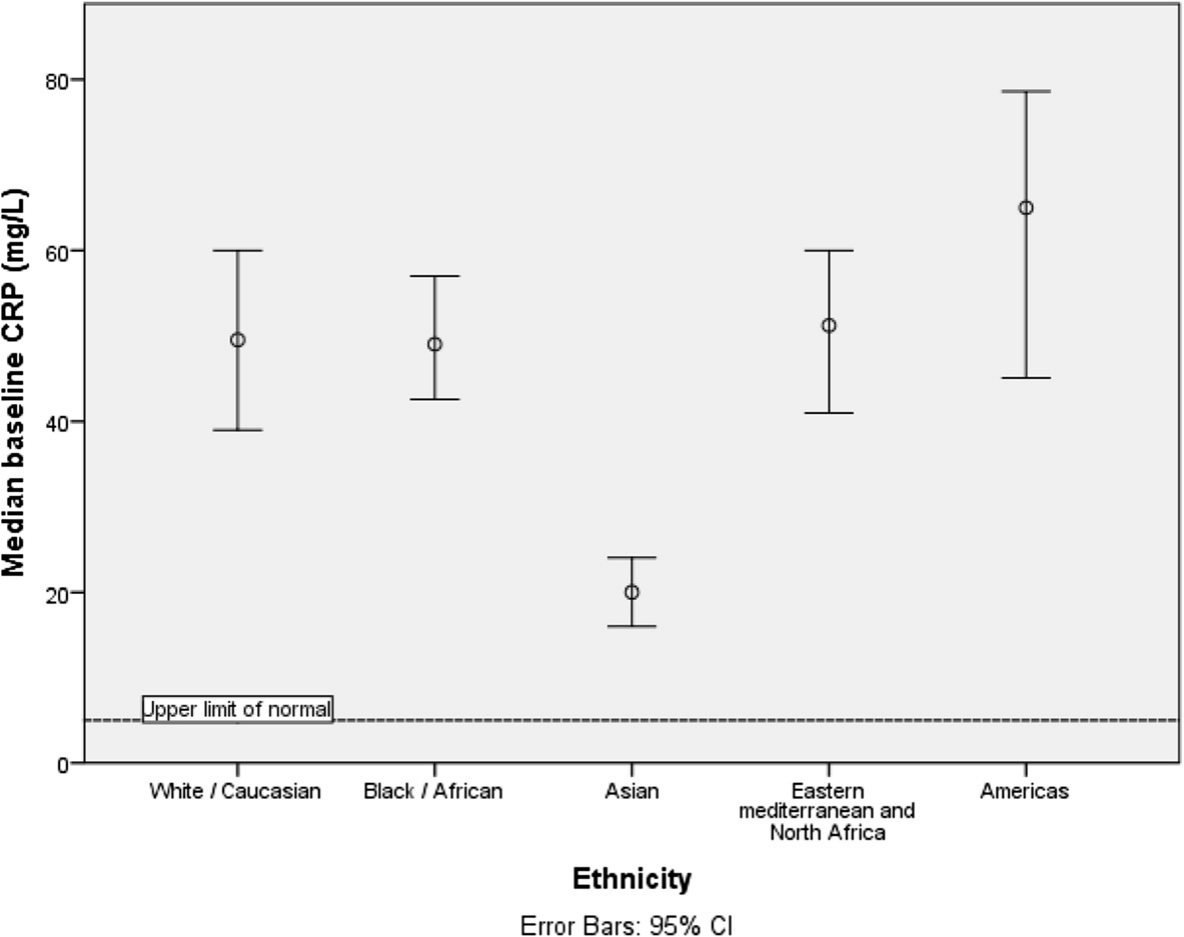
Association between ethnicity and baseline CRP

Disease and patient characteristics were highly associated with baseline CRP (Table 2). The CRP was greater in patients with culture positive disease compared to culture negative (49 mg/L vs 19 mg/L *p* < 0.001). In those with pulmonary disease, smear positive cases had higher CRPs than smear negative subjects (66 mg/L vs 28 mg/L *p* < 0.001). Men had higher baseline CRPs than women (51 mg/L vs 32 mg/L *p* < 0.001) and adults had higher CRPs than children. Significant differences were noted at certain sites of disease, such that pulmonary (median CRP 47 mg/L), pleural or pericardial (84 mg/L) or abdominal (65 mg/L) had higher CRPs than peripheral lymph node (14 mg/L), skin and soft tissue (11 mg/L) or CNS disease (13 mg/L) (Fig. 1). Ethnicity appeared also to influence baseline CRP, with lower values found in those of Asian ethnicity, who had a median CRP of 20 mg/L (Fig. 2).

**Table 2:** Association of clinical parameters with baseline CRP value

		Baseline CRP	
		Median (IQR)	
Age category	Age <16	16 (4–48)	*p* < 0.001*
	Age 16–35	40 (11–93)	
	Age 35–50	50 (14–107)	
	Age >50	39 (13–98)	
Gender male	Male	51 (15–109)	*p* < 0.001**
	Female	32 (11–78)	
Ethnicity	White	50 (16–107)	*p* < 0.001*
	Black African/Caribbean	49 (16–103)	
	Asian	20 (7–54)	
	Eastern Mediterranean and North Africa	51 (17–112)	
	Americas	65 (28–109)	
HIV status	HIV negative	37 (10–88)	*p* < 0.001*
	HIV positive	75 (26–136)	
	HIV status unknown	35 (11–86)	
Smear status	Smear negative	28 (7–74)	*p* < 0.001**
	Smear positive	66 (30–121)	
Culture result	Culture negative	19 (5–72)	*p* < 0.001**
	culture positive	49 (16–103)	
Site of disease	Pulmonary or mediastinal lymph node	47 (14–100)	*p* < 0.001*
	Pleural or pericardial	84 (36–132)	
	Abdominal	65 (29–124)	
	Miliary/disseminated	61 (26–125)	
	Bone and joint	33 (10–78)	
	Peripheral lymph node	14 (4–41)	
	Skin and soft tissue	11 (1–61)	
	CNS	13 (4–31)	
	Other./unknown	33 (12–76)	

*Kruskall Wallis

**Mann Whitney U Test

Many of the characteristics related to baseline CRP were strongly correlated with each other, thus confounding the apparent associations in this univariable analysis, (for instance, pulmonary disease was significantly less common, and peripheral lymph node disease more common, in those with Asian ethnicity). We therefore completed a multivariable analysis (using the log-CRP for linear regression) (Table 3). Here, TB culture status, site of disease and gender remained significantly associated with higher baseline CRP result. Asian ethnicity was significantly associated also with a lower baseline CRP compared to other ethnic groups (Asian ethnicity, adjusted fold-change in CRP 0.49 (95 % CI 0.40–0.58) compared to Black ethnicity).

**Table 3:** Log-scale linear regression model of effect of disease parameters on baseline CRP in active Tuberculosis

		Fold-change in CRP, univariable analysis		Fold-change in CRP, multivariable analysis	
TB culture result	Negative	0.52 (0.46–0.58)	*p* <0.001	0.55 (0.47–0.64)	*p* < 0.001
	Positive	Reference			
Ethnicity	White	0.97 (0.83–1.14)	*p* = 0.737	0.87 (0.81–1.04)	*p* = 0.139
	Americas	1.37 (1.05–1.80)	*p* = 0.022	1.22 (0.89–1.67)	*p* = 0.213
	Asian	0.51 (0.44–0.60)	*p* <0.001	0.49 (0.40–0.58)	*p* < 0.001
	Eastern Mediterranean	1.04 (0.86–1.26)	*p* = 0.7	1.06 (0.85–1.31)	*p* = 0.570
	Black	Reference			
HIV status	Positive	1.77 (1.49–2.10)	*p* < 0.001	1.37 (1.13–1.67)	*p* = 0.001
	Negative	Reference			
Age	<16	0.48 (0.37–0.62)	*p* < 0.001	0.68 (0.46–0.99)	*p* = 0.044
	16–35	0.94 (0.82–1.09)	*p* = 0.427	0.86 (0.70–1.03)	*p* = 0.104
	35–50	1.08 (0.92–1.27)	*p* = 0.351	0.88 (0.72–1.08)	*p* = 0.223
	<50	Reference			
Site of disease	Pleural/pericardial	1.70 (1.40–2.06)	*p* < 0.001	1.49 (1.17–1.88)	*p* = 0.01
	Abdominal	1.56 (1.19–2.06)	*p* = 0.001	1.77 (1.29–2.42)	*p* <0.001
	Miliary/disseminated	1.46 (1.07–2.00)	*p* = 0.018	1.24 (0.86–1.79)	*p* = 0.243
	Bone/joint	0.84 (0.68–1.04)	*p* = 0.111	0.92 (0.71–1.19)	*p* = 0.540
	Peripheral lymph node	0.44 (0.38–0.52)	*p* < 0.001	0.52 (0.42–0.63)	*p* <0.001
	Skin/soft tissue	0.29 (0.18–0.48)	*p* < 0.001	0.41 (0.23–0.70)	*p* = 0.001
	CNS	0.41 (0.31–0.55)	*p* < 0.001	0.38 (0.28–0.53)	*p* < 0.001
	Pulmonary	Reference			
Gender	Male	1.40 (1.24–1.58)	*p* <0.001	1.29 (1.13–1.48)	*p* <0.001
	Female	Reference			

Differences in M.tb strain type have been associated with differing clinical characteristics. For example, the Beijing strain was found in some analyses to relate to increased virulence and acquisition of drug resistance [14, 15]. We therefore sought to test the hypothesis that strain type could be affecting baseline CRP response within our dataset. This was performed in a subgroup of patients for whom data on strain-typing by VNTR were available (routinely conducted since 2010) and undertaken only in cases with pulmonary disease to allow for the strong effect of disease site on baseline CRP and the relatively small numbers with culture-confirmed disease for whom strain-type data might be available at other body sites.

**Fig. 3:**
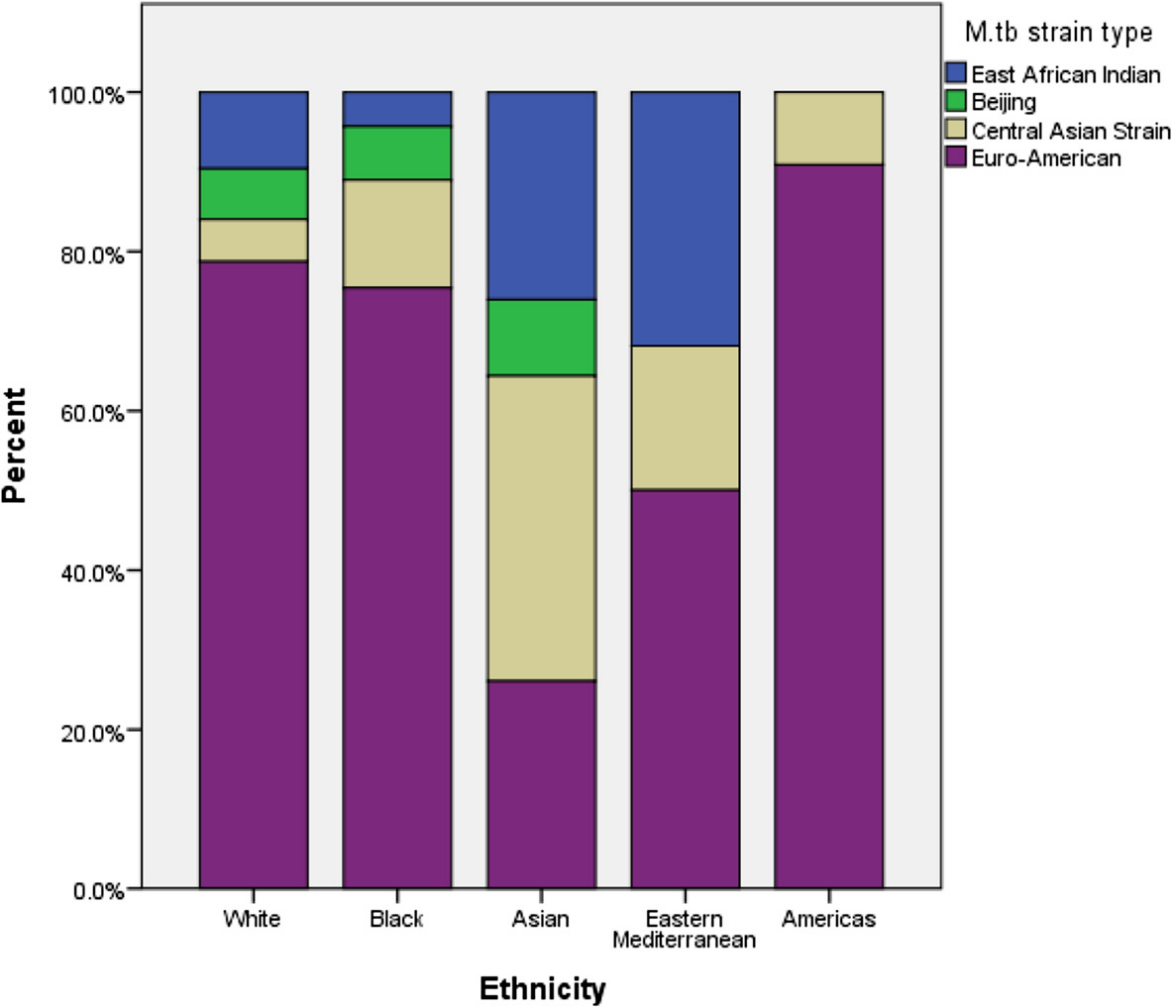
Distribution of different of *Mycobacterium tuberculosis* (M.tb) strain types according to host ethnicity in patients with culture-positive pulmonary tuberculosis

Four hundred and three cases with culture-confirmed pulmonary disease had both strain-type and baseline CRP available, and were included in this analysis. Similar relationships between gender, HIV status, ethnicity and age were present in this subgroup as in the full dataset, albeit with lower power to detect differences given the smaller sample size. Unadjusted analyses suggested lower baseline CRPs for those with East African Indian (EAI) or Central Asian Strain (CAS) type compared to Euro-American (the dominant strain in this population representing 55 % of isolates) or Beijing strains. It should be noted that those of Asian ethnicity were more likely to have an infection with isolates of CAS or EAI strain types (Fig. 3), which tended to be associated with lower CRPs. The apparent association between Asian ethnicity and lower baseline CRP was therefore significantly attenuated in the fully adjusted multivariable model which included strain type, where those of Asian ethnicity had a lower baseline CRP than those of Black ethnicity (the reference category in this analysis): adjusted fold-change in baseline CRP 0.72 (0.53–0.99) (Table 4). White ethnicity was also associated with lower CRPs than Black ethnicity: fold-change 0.71 (0.53–0.95) *p* = 0.020. Strain-type was associated with baseline CRP, although a statistically significant difference was only found with isolates of EAI strain type, where a 0.49 (0.33–0.73, *p* = 0.001) fold lower adjusted baseline CRP was seen compared to those of Euro-American strains.

**Table 4:** Effect of *Mycobacterium tuberculosis* (M.tb) strain type and host ethnicity in patients with culture-positive pulmonary tuberculosis

Parameter		Unadjusted baseline CRP median(IQR) mg/L	Fold change in CRP, multivariable (log-scale) linear regression model*	
M.tb strain type	Beijing	62 (17–82)	0.93 (0.55–1.55)	*p* = 0.772
	East African-Indian	29 (14–70)	**0.49 (0.33–0.73)**	***p*** **= 0.001**
	Central Asian Strain	28 (14–84)	0.71 (0.50–1.00)	*p* = 0.053
	Unassigned	56 (28–115)	0.97 (0.68–1.38)	*p* = 0.871
	Euro-American	70 (30–124)	Reference	
Ethnicity	Americas	59 (22–82)	0.73 (0.37–1.43)	*p* = 0.357
	Asian	30 (15–75)	**0.72 (0.53–0.99)**	***p*** **= 0.045**
	Eastern Mediterranean	73 (40–118)	1.33 (0.81–2.19)	*p* = 0.260
	White	57 (19–113)	**0.71 (0.53–0.95)**	***p*** **= 0.020**
	Black	73 (28–128)	Reference	

*Adjusted for age, gender and HIV status as well the parameters in the table

Factors significant at 0.05 significance level highlighted in bold

Sensitivity analyses were undertaken using different coding strategies. The data on ethnicity depended on that recorded by the treating clinicians (which might be incorrect); using place of birth as a proxy for ethnicity and dividing this into WHO regions (to give 6 categories) resulted in very similar findings, with lower CRPs in those of Southeast Asian and Western Pacific regions in unadjusted analyses. Our full dataset included any patient treated for TB, to allow analysis of culture negative cases. Repeating the analysis with only culture positive cases demonstrated similar associations between the other examined clinical parameters.

## Discussion

This analysis of a large cohort of patients treated for active TB in a low-TB incidence, resource-rich setting demonstrates that the baseline CRP differs considerably with host and disease characteristics. We found significantly lower median CRP results in patients with disease at sites typically associated with low mycobacterial load such as peripheral lymph node or CNS disease [16, 17]. CRP was also lower in HIV negative compared to HIV positive people [18], and culture negative compared to culture positive cases. One hypothesis that would account for these associations is that CRP could reflect mycobacterial load, although we note that pleural and pericardial disease (which are also thought to be paucibacillary) also had high baseline CRPs, which in these cases may reflect a more inflammatory immune response.

We found an apparent association between Asian ethnicity and a lower median baseline pre-treatment CRP. However when we adjusted for site of disease and M.tb strain type, using an analysis restricted to culture positive pulmonary cases, this association was weakened, suggesting that this may be partially explained by infection with either EIA or CAS strains predominating in Asian patients. However, differences in illness behaviours and barriers to access of care between ethnic groups could also act as a confounding factor which could influence baseline CRPs in our cohort.

The serum CRP is well established as a clinically useful biomarker in other conditions - for instance it has been shown to predict outcome and have a role in guiding therapy in community acquired pneumonia [19, 20]. If serum CRP reflects TB mycobacterial load then this would support its use as part of clinical evaluation in active TB. For example, a high baseline CRP might suggest that longer treatment duration is necessary (importantly, a high baseline CRP appears to be associated with an increased likelihood of death in active TB [8]) Equally, the failure of CRP to normalise with treatment may indicate that therapy is less effective than it should be (e.g. due to drug failure or poor adherence) - triggering increased clinical input and re-assessment. This study has evaluated CRP as a blood biomarker, however evaluation of CRP levels in other specimens, such as saliva may also be of value [21] or of other inflammatory biomarkers which might be detectable in urine (which could perhaps be evaluated as point-of-care tests in the future [22]).

Limitations of this study include its retrospective design and the use of routinely collected clinical data. Consequently, only 72 % of patients had a baseline CRP result, and there were significant differences between those with and without a CRP. Given that people in whom the CRP was not measured were more likely to have disease at sites associated here with a low CRP, and less likely to have HIV coinfection, we may have over-estimated the true CRP values for the active TB population as a whole and under-estimated the proportion with a normal CRP level. In addition, differences in the proportion of individuals with a CRP result according to host characteristics (for instance White individuals were less likely to have a CRP measurement) could be a source of bias for our findings. The retrospective design and limited information regarding disease severity mean that we cannot determine if the differences in baseline CRP response seen with M.tb strain type results from greater mycobacterial load being associated with, or resulting from, a particular M.tb strain, or if the strain type itself leads to a different immune response in terms of magnitude or quality to a given number of mycobacteria.

Different bacterial strain types are known to have important effects on clinical presentation and outcomes in other infections, such as *Streptococcus pneumoniae*. [23] It is less clear whether or not significant differences exist between strain types of Mtb although it has been suggested that the Beijing strain is more virulent or more likely to acquire drug resistance [24]. Our data cannot assess this, although encourage further work in this area.

## Conclusions

This study demonstrates that baseline CRP reflects important clinical parameters in patients with active TB and confirms that variation in this immune response occurs with different TB strain-types after adjustment for disease and host characteristics. As well as aiding the interpretation of such baseline CRP results in clinical practice, this lends support to the use of this test as a marker of disease severity in TB.

## Abbreviations

CAS, Central Asian Strain; CNS, central nervous system; CRP, C-reactive protein; EIA, East African Indian (TB strain-type); HIV, human immunodeficiency virus; M.Tb, mycobacterium tuberculosis; TB, tuberculosis

## Additional file

Additional file 1: Table S1. Comparison of cases with and without a CRP result (DOCX 15 kb)
