# Supplementary material for: Variation in C - reactive protein response according to host and mycobacterial characteristics in active tuberculosis
Source: BMC Infect Dis. 2016 Jun 10;16:265. doi: 10.1186/s12879-016-1612-1 (PMC4901496; doi:10.1186/s12879-016-1612-1)
Supplement: Additional file 1: Table S1. — Comparison of cases with and without a CRP result (DOCX 15 kb) [file 12879_2016_1612_MOESM1_ESM.docx]

Table S1 – comparison of cases with and without a CRP result:

|  | | Baseline CRP taken  (n = 2307) | No CRP result  (n = 915) |  |
| --- | --- | --- | --- | --- |
| Age, mean (SD) years | | 38 (17) | 35 (18) | p = 0.01 |
| Culture positive n (row n%) | | 1590 (74.4%) | 546 (25.6%) | p <0 .001 |
| Smear positive (pulmonary cases, total n=515)  n (row n%) | | 424 (82%) | 91 (18%) | p < 0.001 |
| Gender n (row n%) | Male | 830 (67.8%) | 395 (32.2%) | p = 0.035 |
|  | Female | 569 (63.4%) | 329 (36.6%) |  |
| Ethnicity n (row n%) | White / Caucasian | 362 (58.8%) | 254 (41.2%) | p < 0.001 |
|  | Black / African | 705 (72.2%) | 272 (27.8%) |  |
|  | Asian | 465 (72.5%) | 176 (27.5%) |  |
|  | Eastern Mediterranean and North Africa | 221 (74.4%) | 76 (25.6%) |  |
|  | Americas | 97 (65.5%) | 51 (34.5%) |  |
| HIV status n (row n%) | HIV negative | 1562 (77.3%) | 458 (22.7%) | p < 0.001 |
|  | HIV positive | 271 (81.4%) | 62 (18.6%) |  |
|  | HIV status unknown | 472 (54.6%) | 393 (45.4%) |  |
| Site of disease  n (row n%) | Pulmonary and MLN | 1270 (71.3%) | 510 (28.7%) | P <0.001 |
|  | Pleural / pericardial | 192 (82.4%) | 41 (17.6%) |  |
|  | Abdominal | 91 (76.5%) | 28 (23.5%) |  |
|  | Miliary / disseminated | 68 (90.7%) | 7 (9.3%) |  |
|  | Bone and joint | 163 (82.7%) | 34 (17.3%) |  |
|  | Peripheral lymph node | 331 (62.1%) | 202 (37.9%) |  |
|  | Skin and soft tissue | 26 (83.9%) | 5 (16.1%) |  |
|  | CNS | 83 (65.9%) | 43 (34.1%) |  |
|  | Other / unknown | 83 (64.8%) | 45 (35.2%) |  |
